# Flawed prosthodontic treatment as a triggering factor of orofacial dyskinesia: A case report

**DOI:** 10.4317/jced.61635

**Published:** 2024-06-01

**Authors:** Javier Salinas, Bárbara Bello, Camila Antúnez, Diego De Nordenflycht

**Affiliations:** 1 DDS. Instructor, Faculty of Dentistry, Universidad Andres Bello, Viña del Mar, Chile; 2 DDS. Private practice. Viña del Mar, Chile; 3 DDS, MSc. Associate Professor, Faculty of Dentistry, Universidad Andres Bello, Viña del Mar, Chile

## Abstract

Orofacial dyskinesia (ODk) is an involuntary, repetitive and stereotyped movement disorder of the oro-bucco-lingual muscles, which can be classified as primary (idiopathic) or secondary to medical conditions such as oral peripheral factors, that may act as triggers or aggravators. The present case describes a 70 years female with ODk, non-associated to drug use, without central etiological factors or morbid conditions, but with the presence of a flawed prosthodontic treatment, which complaint from spasms in the masticatory muscles that alters jaw dynamics, and her ability for maintain a relaxed jaw in maximal intercuspal position. After an unsuccessful oral drug treatment, botulinum toxin was injected to the jaw muscles with favorable results. The case illustrated that peripheral factors, such as defective dental prosthetics, may trigger or aggravate orofacial movement disorders, and peripheral strategies such as botulinum toxin may contribute to improve clinical parameters and quality of life.

** Key words:**Botulinum toxin, case report, dyskinesia, movement disorder, orofacial dyskinesia.

## Introduction

Orofacial dyskinesia (ODk) is an involuntary, repetitive, and stereotyped movement disorder of the oro-bucco-lingual muscles and may include twisting and protruding movements of the tongue, lip pursing, facial grimacing, and chewing movements (1). Affected subjects might present a diverse clinical phenomenology in terms of complexity and severity, which may vary from almost imperceptible clinical symptoms to a significant deterioration in the quality of life (2). It can also be an infrequent source of painful symptoms of the muscles, joint, or traumatic oral injuries (3). ODk can be spontaneous (primary) or secondary to drug-induced reactions, or tardive syndromes, but can also be secondary to subcortical infarcts, peripheral factors (such as edentulism or ill-fitting dentures), or be concomitant with neuropsychiatric conditions, dementia, or mental retardation (1). It is hypothesized that peripheral oral factors could play an important role in inducing an ODk, although the exact mechanism is still unclear, probably due to a lack of research in the area (4). The association between ODk and peripheral oral factors requires further investigation, in order to identify the exact role that these factors play. This paper aims to discuss the dental management of a patient where oral factors acted as triggers for a peripheral-induced secondary ODk.

## Case Report

A 70-year-old female subject presented the main complaint of involuntary movements of the masticatory muscles, which generate altered jaw dynamics (i.e. mouth opening and closing movements and maintain a relaxed jaw location in maximal intercuspal position), orofacial pain, and a disturbed emotional state. The patient’s medical record included smoking habit (10 cigarettes per day), and history of surgical treatment of one year ago of both hips Osteochondritis dissecans for which she occasionally uses Celecoxib 200 mg/day for painful hips. In March 2023, the patient consulted a neuropsychiatrist, who after a clinical evaluation and complementary exams (brain CT and blood tests, whose results were within normal parameters) discard central degenerative diseases, demyelinating or sclerotic lesions of the nervous system. The initial diagnosis was orofacial dyskinesia and initiated oral drug therapy with Clonazepam 0.5 mg twice a day with unsuccessful results, for which the patient was referred to an orofacial pain dental specialist.

**Figure 1:**
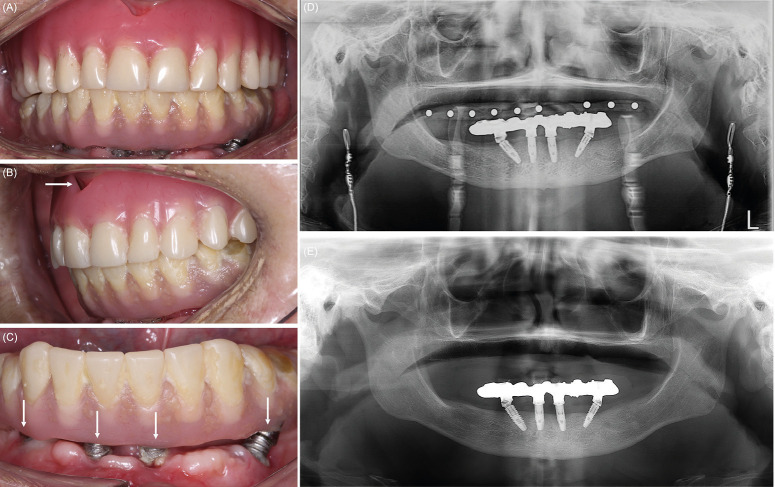
(A) frontal view show a upper complete removable denture, and a lower implant-supported fixed complete hybrid denture; (B) lateral show an ill-fitting denture with a notorious gap (horizontal white arrow) between the residual ridge and the denture base; (C) close-up view of the implant-supported fixed denture showing exposure of the implants platforms and threads (vertical white arrows), dental plaque around implants, and a notorious gap between lower prosthesis and residual mucosa; (D) immediate after-treatment and (E) current panoramic radiographs that evidence a generalized horizontal bone loss and signs of periimplantitis.

According to the patient, the symptoms began immediately after completion of an extensive prosthodontic treatment in August 2021, that involved the replacement of the upper denture, and the installation of a lower implant-supported denture. The case was characterized by an ODk in mouth closing, with presence of repetitive and involuntary movements of the masticatory muscles during jaw movements; also, the patient complained of jaw muscles pain, presumably secondary to ODk. In the clinical exam the patient presented complete edentulism in both the maxilla and mandible, and wearing of an upper complete removable denture and a lower implant-supported fixed complete hybrid denture (Fig. 1A). Both prostheses present defective adjustment, support, stability and occlusion (Fig. 1B,C); the comparison of the after-prosthodontic-treatment and current state panoramic radiographs (20 months apart) evidence a generalized horizontal bone loss of the mandibular alveolar processes and signs of periimplantitis (Fig. 1D,E). Causes for the prosthodontic treatment failure are beyond the scope of this paper. On the basis of the patient’s medical record, brain imaging, blood test, dental radiograph and examination, the diagnosis of orofacial dyskinesia secondary to peripheral factors was confirmed, and other causes for the involuntary jaw movements were ruled out, such as tardive dyskinesia, oromandibular dystonia, drug-induced extrapyramidal reactions, awake bruxism, and Parkinson’s disease. Considering dental features and the poor response to oral medication, it was decided to manage the case with botulinum toxin type-A (BoNT-A) injection in order to control the involuntary movements prior to a new prosthodontic treatment.

**Figure 2:**
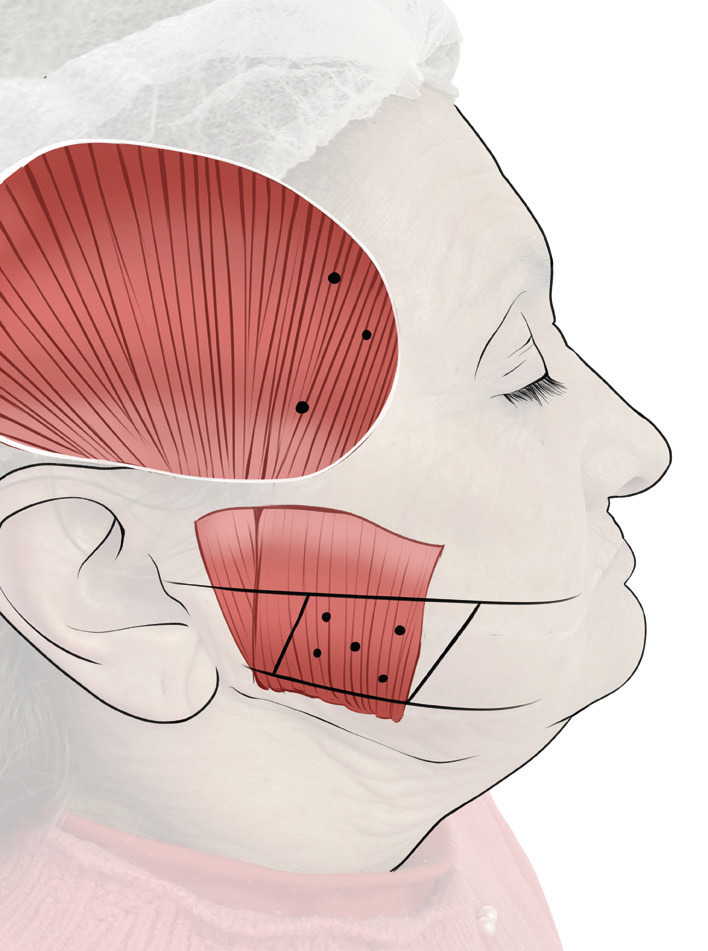
Scheme of the BoNT-A injection points. For the masseter muscle, a rhomboid area was delimited based on a upper line traced from tragus to corner of the mouth, a lower line 5 mm above the mandibular border, and two vertical lines following the anterior and posterior contours of the superficial portion of the masseter muscle; five points of injection with a separation of 5 mm between them were determined, and a total of 30 U of BoNT-A was injected in each masseter muscle. For the temporal muscle, a semicircular line following the anterior contour of the muscle until hairline in the temple region; three points of injection with a separation of 5 mm between them were determined, and a total of 15 U was injected in each temporalis muscle.

**Figure 3:**
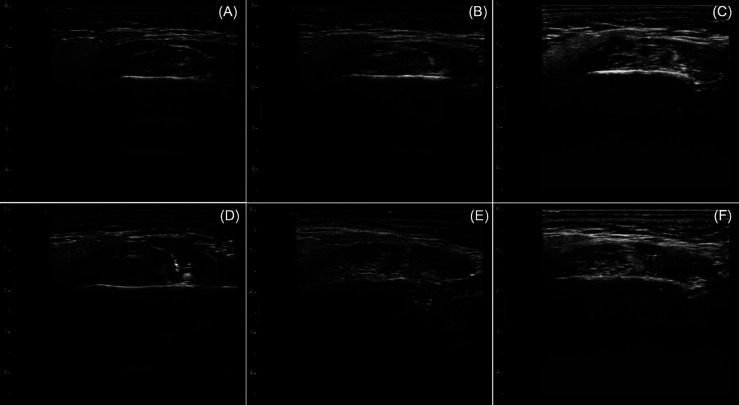
Ultrasonographic captions of the right (A, B, and C) and left (D, E, and F) masseter muscles in axial plane standardized at 2 cm above the mandibular border before treatment (A and D), at 2 weeks after treatment (B and E) and at 4 weeks after treatment. Images show a decrease in muscle thickness, and a change in the intramuscular ultrasonographic appearance.

A total of 90 U of BoNT-A (100 U; Botox®, Allergan, Irvine, CA, USA) was injected to the both sides masseter (30U each side) and temporalis muscles (15U each side). The anatomical references and injection sites are shown in Figure 2. The 100 U vial was reconstituted with sterile saline solution and injected using 1 ml syringe and 30-gauge needle. The patient was asked to clench their teeth to delimit each muscle contour (superficial masseter and anterior temporalis) and then relax the muscle. A total of five injection points in each masseter muscle and three injection points in each temporalis muscle with a separation of 5 mm between them were applied, performed in a single appointment. Clinical and ultrasonographic (12 Mhz linear probe) assessments were performed at 2 and 4 weeks after BoNT-A injection (Fig. 3; additionally, supplementary USG clip is available at osf.io/2y8n7), where it was noticed a considerable decrease in the involuntary mouth movements, absence of jaw muscles pain, and a decrease in mandibular instability during mouth opening and closing movements. Initially, the patient reported emotional compromise due to the functional limitation generated by the movement disorder, as it was evidenced by DC/TMD axis II instruments such as PHQ-9=13 (moderate depression), GCPS=IV (severely limiting) and JFLS-20=9.15. One month after treatment, psychometric parameters improvement was observed for PHQ-9=7 (mild depression), GCPS=III (moderately limiting) and JFLS-20=4.37. Non side effects were reported by the patient. Finally, the patient was referred to a prosthodontic specialist for denture replacement and dental implants maintenance.

## Discussion

The present case illustrates a clinical presentation of a secondary peripheral-induced orofacial dyskinesia in which a flawed prosthodontic treatment acted as a triggering factor. It has been stated that in the absence of other putative factors, edentulism and orodental problems may induce an ODk (5). ODk can be classified as primary (spontaneous, or idiopathic) or secondary to drug-induced reactions (or tardive syndromes), subcortical infarcts, or peripheral factors (1). The overall prevalence was estimated at 3.7% (4.1% for females and 2.9% for males) (5). Complications of ODk may include tooth wear and fracture, prosthesis damage and displacement, accelerated bone loss among edentulous patients, orofacial pain, temporomandibular joint degeneration, ulcers secondary to tongue and cheek biting, dysarthria, dysphagia, chewing difficulties, inadequate food intake and weight loss, and social embarrassment secondary to compromised facial esthetics (1,6).

Oral peripheral factors such as edentulism, poorly fitting removable dentures, oral pain or discomfort, and poor perceived oral health appear to be strongly associated in some ODk cases, but the exact mechanism remains unclear (5). Although edentulism appears to be a common cause of ODk, the number of studies on this association is low (1). A cross-sectional study involving 1,018 subjects found that patients with ODk more frequently reported poorly fitting dentures, oral pain, and poor perception of oral health (5). In contrast to drug-induced dyskinesia, the abnormal movements observed in peripheral-induced ODk are confined to the oral region and never dystonic, excluding the tongue when the mouth is open (7). Also, edentulism has also been associated with higher scores of tardive ODk compared to dentate patients (8). It has been postulated that cortical reorganization in response to altered peripheral inputs could be the mechanism underlying these peripherally induced movement disorders (9).

Management of orofacial movement disorders can be divided into medical management (with centrally acting drugs such as benzodiazepines, anticholinergics, GABAergics, dopaminergics, and antiparkinsonians), chemodenervation using BoNT-A, and surgical management (6). To date, there are no controlled trials that reported the use of medications in edentulous patients with ODk (7), so its use is still an experience-based practice. Chemodenervation with BoNT-A has become the modality of choice for patients who have orofacial movement disorders because of its high-efficacy, albeit temporary relief (6). Its therapeutic benefit is mainly attributable to its action of blocking the release of acetylcholine into the neuromuscular junction by cleaving SNARE proteins that are required for the docking of the acetylcholine vesicle to the presynaptic membrane (10). It has been also postulated that some clinical effects could be centrally mediated (11), however, further research is necessary to establish a causal relationship between clinical improvement and the central effects of BoNT-A.

Finally, given that neurological, psychiatric, and dental factors may contribute, to a variable extent to ODk, an interdisciplinary assessment involving a neurologist, psychiatrist, and dentist is recommended for comprehensive management (12). From a clinical point, BoNT-A injection might be needed in ODk edentulous patients prior to the beginning of prosthodontic treatment given that oromandibular movement disorders might interfere with the determination of the maxillomandibular relationship and the occlusal vertical dimension.

## Data Availability

The datasets used and/or analyzed during the current study are available from the corresponding author.
